# Experience-related reductions of myelin and axon diameter in adulthood

**DOI:** 10.1152/jn.00070.2018

**Published:** 2018-09-12

**Authors:** Alberto Lazari, Sigrid Koudelka, Cassandra Sampaio-Baptista

**Affiliations:** ^1^Wellcome Trust Doctoral Programme in Neuroscience, University of Oxford, Oxford, United Kingdom; ^2^Wellcome Centre for Integrative Neuroimaging, FMRIB, Nuffield Department of Clinical Neurosciences, University of Oxford, Oxford, United Kingdom; ^3^Centre for Discovery Brain Sciences, University of Edinburgh, Edinburgh, United Kingdom

**Keywords:** auditory processing, conduction velocity, myelination, plasticity, sensory deprivation

## Abstract

The production of new myelin has been highlighted as an underappreciated mechanism of brain plasticity, but whether plastic decreases in myelin also happen in the adult brain has been largely unexplored. Recently, Sinclair et al. (Sinclair JS, Fischl MJ, Alexandrova O, Heß M, Grothe B, Leibold C, and Kopp-Scheinpflug C. *J Neurosci* 37: 8239–8255, 2017) have shown that auditory deprivation can lead to decrease in myelination and axon caliber even in healthy adulthood. These findings show that activity-regulated myelination is more complex than previously thought and expand our knowledge of how adult brain plasticity could operate on a cellular level.

Myelination of axonal pathways has long been recognized as necessary for enhancing action potential conduction within the central nervous system and thus essential for healthy brain function. However, plastic changes in myelin during adulthood have only recently started being widely reported. How, when, and why such changes occur remains to be fully understood. Nevertheless, the emerging concept of adaptive myelin plasticity is revealing an additional and complementary mechanism to synaptic plasticity by which timing in neural circuits can be dynamically regulated throughout life.

Comparing adaptive myelin changes with synaptic plasticity provides a useful framework by which to expose our lack of knowledge about the rules governing myelin plasticity (Fields 2015). For example, we know that synaptic plasticity is bidirectional (i.e., synapses can undergo potentiation and de novo formation, as well as depression and pruning), but few studies have examined whether myelin can also undergo decrements as well as increments in its morphological features. Recently, there has been accruing evidence from in vivo studies in the mouse optic nerve (Etxeberria et al. 2016) and in the zebrafish spinal cord (Mensch et al. 2015) that there are decreases in myelination in the absence of neuronal activity. Yet these studies have often been restricted to developing systems, which are particularly sensitive to reduced stimulation.

Reports of reduction in myelination in the healthy adult brain have been sparse and typically associated with stressful conditions such as social deprivation or defeat, where the underlying changes in neuronal activity are complex (Lehmann et al. 2017). Hence, further demonstrations of decreases in myelin thickness would be crucial to the understanding of myelin plasticity mechanisms in the adult brain.

In a recent study published in *The Journal of Neuroscience*, Sinclair and colleagues (2017) reveal that sensory deprivation can lead to myelination reductions in healthy adult mice. The study aimed to characterize the structural and functional development of myelinated axons in the auditory system and to probe how deprivation of auditory stimuli affects myelin and axonal processes. The results mapped the developmental trajectory of the trapezoid body and identified serial peaks in conduction velocity, axon diameter, and myelin thickness (at postnatal days ~P18, ~P25, and ~P30, respectively).

One key experiment from the paper reveals important insights into how myelin plasticity is shaped by sensory activity. In this paradigm, adult P65–75 mice were deprived of auditory stimuli by inserting foam earplugs into the external auditory meatus. At the end of the 10 days, immunohistochemistry was used to measure the diameter of trapezoid body axons and the thickness of their myelin sheaths. The results (summarized in Fig. 1*B*) revealed that adult sensory deprivation leads to a decrease in the number of large, auditory trapezoid body axons, and a reduction in myelin sheath thickness specifically on these axons. This important observation indicates for the first time that sensory deprivation in adulthood can result in reduced myelination and axonal caliber.

**Fig. 1.**
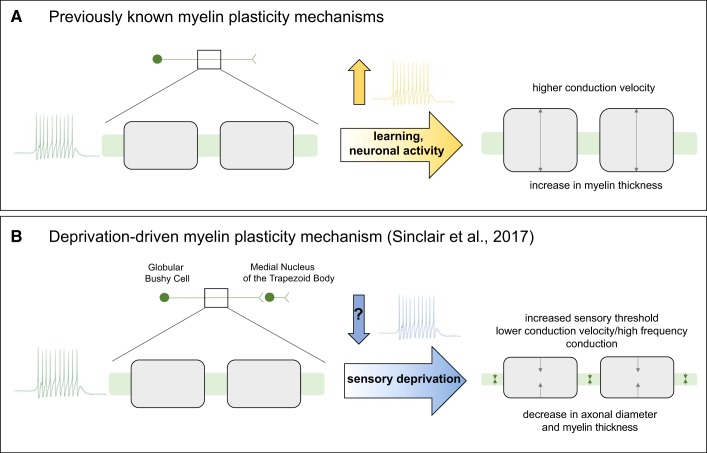
Neuronal activity and experience may bias myelination bidirectionally. Schematic of two complementary mechanisms by which neuronal activity putatively shapes myelination. *A*: previous literature has largely focused on increases in myelination. Several strands of evidence suggest that both increases in neuronal activity and experience (such as motor learning) may drive such increases in myelin during adulthood. However, the question remains open whether axonal diameter is regulated in a similar manner. *B*: summary of the deprivation-driven mechanism described by Sinclair and colleagues (2017). The results suggest that auditory globular bushy cell projections in the auditory brain stem (*left*) are affected by sensory deprivation, which leads to decreases in their myelin thickness and axonal diameter (*right*). Though it is foreseeable that sensory deprivation may lead to an overall decrease of neuronal firing and that the deprivation-driven response may be inactivity dependent, the specific pattern of neuronal activity triggered by auditory deprivation was not explored.

As outlined in Fig. 1*A*, studies on activity-dependent myelination have, until recently, focused on the plastic potential of myelin sheath properties rather than that of the axon diameter. There are few exceptions—notably, a recent paper by Chéreau et al. (2017). This study applied stimulated emission depletion microscopy to resolve axonal morphology in hippocampal slices and reported a long-term increase in axon diameter following high-frequency neuronal firing. However, whether the same mechanism would apply in vivo and to behavioral paradigms known to induce myelin plasticity is unknown. For instance, it has not been explored whether axon diameter changes during learning as myelin does. Because fractional anisotropy, a commonly used marker of white matter microstructure in humans, increases with both increases in myelination and decreases in axonal diameter, axonal plasticity could also be compatible with multiple results showing learning-related and experience-related changes in diffusion metrics in humans (for a review, see Sampaio-Baptista and Johansen-Berg 2017) including alterations in the corticospinal tract following two weeks of sensorimotor deprivation (Langer et al. 2012).

This makes it all the more striking that axonal changes were observed in the study (Fig. 1*B*) and prompts many questions about the nature of the reported deprivation-driven response. One possibility is axonal degeneration. The study does not examine whether mice undergoing sensory deprivation show higher levels of apoptotic proteins — therefore, the observed fall in large diameter axon percentage might have involved cell death and/or axonal degeneration. On one hand, this possible axonal loss or alteration could be a primary mechanism. The brief induction of midazolam/medetomidine/fentanyl (MMF) anesthesia to allow earplug insertion was not matched in the sham group; since anesthetics with similar mechanisms of actions to MMF can have neurodegenerative effects (Jevtovic-Todorovic et al. 2003), anesthesia-induced axonal degeneration cannot be ruled out. On the other hand, axonal loss could be a secondary effect of the reduction in neuronal activity or could stem from the reduction or degeneration of myelin, as myelin is important for maintaining axonal integrity (Lee et al. 2012b).

Dynamic regulation of axon diameter, rather than axonal degeneration, may also play a major role in this and other experiments investigating myelin modulation. As axon caliber is an important regulator of myelination (Lee et al. 2012a), reductions in myelin could be explained as being induced by a decrease in axon caliber. In this framework, an alternative interpretation of the study’s results is that the globular bushy cell axons simply shrank, leading to the myelin changes described. The paper indicates that the percentage of large axons decreases significantly, but it is not stated whether this is coincidental with an increase in the percentage or absolute number of small diameter axons. In addition, the g-ratio (the ratio of inner axon diameter to total outer diameter of the axon diameter, including the myelinated sheath) is widely acknowledged as a physiologically relevant metric to quantify myelinated axons and could easily complement axon and myelin morphology measures to clarify the physiological relevance of the observed changes. For these reasons, future analyses in this and other myelin plasticity experiments could provide more detailed explanations by comparing *1*) the overall distribution of axon diameters and *2*) the g-ratio between conditions.

How do these deprivation-driven reductions in adult myelination fit with the current understanding of myelin plasticity? The underlying cellular mechanisms of myelin growth are still controversial, and potential mechanisms for myelination reductions remain similarly opaque. In the simplest and most likely scenario, oligodendrocytes might integrate information from reduced or absent axonal activity and respond by actively retracting or shortening their myelin sheaths. However, exactly how such communication between the axon and the myelin sheath takes place is not fully understood. The signaling pathways within oligodendrocytes that may mediate myelin sheath reductions are also not fully understood. As identified by Sinclair and colleagues, multiple molecular candidates, such as the ERK1/2 pathway (Jeffries et al. 2016), have been implicated in increased myelin thickness; but whether the same pathways are involved in thickness decreases remains unexplored.

As evidence for myelin plasticity accumulates from studies of a variety of brain regions, it becomes increasingly pressing to understand whether these mechanisms are universal across neuroanatomical domains or whether they are specialized. It is tempting to see the results from Sinclair and colleagues as strongly anatomically specific. In the trapezoid body, precisely timed conduction of action potentials is particularly crucial. Therefore, it is plausible that the auditory brain stem has evolved mechanisms to tightly regulate conduction velocity of its axons. Interestingly, a previous study by Etxeberria et al. (2016) addressed the existence of a similar deprivation-driven mechanism in the visual system, though during development rather than adulthood. The study examined the effect of monocular deprivation in the optic nerve and found a decrease in conduction velocity similar to that in Sinclair et al. (2017). However, the two studies highlighted divergent mechanisms to explain the result. While in Sinclair et al. (2017) the auditory axon diameter and myelin thickness were reduced (Fig. 1*B*), in Etxeberria et al. (2016) the optic nerve axon and myelin morphology was kept constant, but an internode size reduction was observed. This suggests that myelin and axonal regulation may be anatomically diverse and could exhibit both convergent and divergent features in other sensory and motor domains.

A second question regarding myelin plasticity is whether it can actively shape behavior and cognition. Does the mechanism described by Sinclair et al. (2017) play a causal role in influencing sensory perception following sensory deprivation? Increased sensory thresholds in ear plugged animals were reported, but such findings could also be attributable to concurrent synaptic plasticity driven by the same sensory deprivation. The low sample sizes (3 mice per group) precludes conducting further analyses such as statistical correlations between the observed histological changes and behavioral variables. For example, having larger sample size could have clarified whether the amount of myelin thickness and/or axon caliber reduction shown in each individual mouse corresponded with the magnitude of auditory threshold elevation. Follow-up studies will likely test whether myelin changes correlate with the observed behavioral changes.

A third question relates to how myelin and axonal plasticity coexists with additional forms of plasticity in the myelinated axon. Like myelin thickness and axon diameter, a recent study by Arancibia-Cárcamo et al. (2017) suggested that Nodes of Ranvier (NoR) might also regulate features of an axon’s neurophysiology. The article by Arancibia-Cárcamo and colleagues highlighted that the length of NoR is highly heterogeneous, but less variable within the same axon than between different axons, suggesting that NoR length may be regulated in a axon-specific manner. Moreover, the study used simulations to show that changes in NoR length may allow adjustment of axon conduction velocity with a smaller change in membrane area, and thus in a more energy-efficient manner, compared with myelin sheath changes. Given previous evidence that node diameter increases progressively in distal axon segments in the trapezoid body (Ford et al. 2015), the contribution of nodal features is especially likely to be relevant to the axon and myelin-related changes reported by Sinclair and colleagues (2017). The cross-sectioning of the axons in the study prevented nodal analyses. However, the results leave room to speculate that modifications of NoR may coexist and act synergistically with myelin and axon caliber changes.

This growing array of brain plasticity mechanisms (involving myelin, axons, and nodes) raises a fourth question: what are the specific functions of these regulatory mechanisms? It is possible that multiple changes in the myelinated axon may act in concert to fine-tune neurophysiological circuit properties. The simulations carried out in the Sinclair et al. (2017) paper tested this hypothesis. The results show that although reductions in axon diameter and myelin thickness both lead to decreased conduction velocity, the two interact nonlinearly to determine the conduction velocity of the axon. Therefore, seemingly simple changes in electrical properties with age and sensory experience can arise from concurrent plasticity of these two biophysical properties. An additional and complementary possibility is that different plasticity mechanisms may prevail depending on the level of energetic demand or on the timescale involved. Although it was beyond the scope of the paper to investigate this, the phenomenon described by Sinclair and colleagues provides an appealing model with which these hypotheses could be tested in the future.

Besides delivering key indication of a new form of experience-dependent myelin plasticity in adulthood, the evidence presented by Sinclair et al. (2017) is particularly timely as it brings to the spotlight the need for myelin plasticity experiments to carefully address the potential roles of axon diameter modifications. In this respect, the study nicely complements a nascent literature exploring the contribution of nonmyelin components, such as features of NoR, to dynamic changes in the myelinated axon (Arancibia-Cárcamo et al. 2017; Etxeberria et al. 2016). This expanding range of plasticity mechanisms is a vital contribution to our understanding of how adult brain plasticity could operate on a cellular level and sets the stage for further investigations into the circuit-level and behavioral relevance of such plastic changes, and the molecular mechanisms that could underlie them.

## GRANTS

A. Lazari is funded by a PhD Studentship from the Wellcome Trust (grant number 109062/Z/15/Z).

## DISCLOSURES

No conflicts of interest, financial or otherwise, are declared by the authors.

## AUTHOR CONTRIBUTIONS

A.L. prepared figures; A.L. drafted manuscript; A.L., S.K., and C.S.-B. edited and revised manuscript; A.L. and C.S.-B. approved final version of manuscript.
